# Multi-Center Randomized Phase II Clinical Trial on Remote Ischemic Conditioning in Acute Ischemic Stroke Within 9 Hours of Onset in Patients Ineligible to Recanalization Therapies (TRICS-9): Study Design and Protocol

**DOI:** 10.3389/fneur.2021.724050

**Published:** 2021-11-03

**Authors:** Susanna Diamanti, Simone Beretta, Mauro Tettamanti, Simona Sacco, Giuliano Sette, Raffaele Ornello, Cindy Tiseo, Valeria Caponnetto, Mario Beccia, Diletta Alivernini, Rocco Costanzo, Carlo Ferrarese

**Affiliations:** ^1^Stroke Unit and Neurology Unit, Azienda Socio Sanitaria Territoriale (ASST)-Monza San Gerardo Hospital, University of Milano-Bicocca, Monza, Italy; ^2^Dipartimento di Ricerca Neuroscienze, Istituto di Ricerche Farmacologiche Mario Negri Istituti di Ricovero e Cura a Carattere Scientifico (IRCCS), Milano, Italy; ^3^Department of Applied Clinical Sciences and Biotechnology, University of L'Aquila, L'Aquila, Italy; ^4^NEuroscienze Salute Mentale e Organi di Senso (NESMOS) Department, Faculty of Medicine and Psychology, Sant'Andrea Hospital, Sapienza University of Rome, Roma, Italy

**Keywords:** acute stroke, ischemic stroke, remote ischemic conditioning, RIC, phase II, clinical trial, TRICS-9

## Abstract

**Aim:** To assess the efficacy of remote ischemic conditioning (RIC) in patients with ischemic stroke within 9 h of onset, that are not candidates for recanalization therapies.

**Sample Size Estimates:** A sample size of 80 patients (40 in each arm) should yield 80% power to detect a 20% difference in early neurological improvement at 72 h at *p* = 0.05, two sided.

**Methods and Design:** TRICS-9 is a phase II, multicenter, controlled, block randomized, open-label, interventional clinical trial. Patients recruited in Italian academic hospitals will be randomized 1:1 to either RIC plus standard medical therapy or standard medical therapy alone. After randomization, RIC will be applied manually by four alternating cycles of inflation/deflation 5 min each, using a blood pressure cuff around the non-paretic arm.

**Study Outcomes:** The primary efficacy outcome is early neurological improvement, defined as the percent change in the National Institute of Health Stroke Scale (NIHSS) at 72 h in each arm. Secondary outcomes include early neurologic improvement at 24 and 48 h, disability at 3 months, rate of symptomatic intracerebral hemorrhage, feasibility (proportion of patients completing RIC), tolerability after RIC and at 72 h, blood levels of HIF-1α, and HSP27 at 24 h and 72 h.

**Discussion/Conclusion:** RIC in combination with recanalization therapies appears to add no clinical benefit to patients, but whether it is beneficial to those that are not candidates for recanalization therapies is still to be demonstrated. TRICS-9 has been developed to elucidate this issue.

**Clinical Trial Registration:** ClinicalTrials.gov, identifier: NCT04400981.

## Introduction and Rationale

Remote ischemic conditioning (RIC) relies on a transient ischemia being applied to a certain body site with the aim of increasing ischemic tolerance in distant organs by endogenous protective mechanisms (1–3). Although these mechanisms remain elusive, evidence supports the role of both humoral and neuronal factors, such as the release of adenosine, bradykinin, and nitric oxide in the blood, and the activation of neuronal p-AKT and several miRNAs (4). Recent observations have suggested that HSP27 and HIF-1α are mediators of remote ischemic conditioning and hence potential biomarkers (5–12). Many experimental studies on animal models of ischemic stroke have demonstrated that RIC is neuroprotective against cerebral ischemia and that benefit is obtained in stroke models with or without reperfusion up to 6 h after stroke induction (13–19). Despite numerous preclinical studies, clinical evidence is limited. Clinical studies showed that remote ischemic conditioning, applied by transient limb ischemia using a blood pressure cuff, is well-tolerated, feasible, and safe (20, 21) and that long-term treatment decreases the recurrence risk of stroke due to symptomatic intracranial arterial stenosis (22). Two clinical trials on RIC in combination with intravenous thrombolysis or mechanical thrombectomy gave neutral results (23, 24); however, no studies have investigated whether RIC is beneficial to patients with acute ischemic stroke who are not candidates for recanalization therapies. These patients represent a significant proportion of those with acute ischemic stroke and although criteria for patient eligibility are continuously evolving, the proportion of ischemic strokes potentially eligible for endovascular thrombectomy or intravenous thrombolysis is limited (25, 26).

The Italian Stroke Organization (ISO) Basic Science Network, a nationwide network that promotes translational research on acute ischemic stroke, has launched a multicenter translational research program on RIC. This program provides for a multi-center pre-clinical study of experimental ischemic stroke (27) and a multi-center randomized phase II clinical trial on remote ischemic conditioning in patients with acute stroke within 9 h of onset who are not candidates for recanalization therapies (TRICS-9).

The TRICS-9 trial is designed to investigate the efficacy of RIC in a carefully selected population of ischemic stroke patients whose neurological improvement could be attributed to no acute treatments but RIC, to evaluate the potential role of RIC as acute therapy for patients that are not a candidate for recanalization therapies and who would otherwise receive secondary prevention only.

The study protocol of the clinical trial is described hereafter.

## Methods and Analysis

### Design

The study is a phase II, multicenter, controlled, block randomized, open-label, interventional clinical trial comparing RIC plus standard medical therapy to standard medical therapy alone, in patients with acute ischemic stroke within 9 h of onset that are not eligible to intravenous thrombolysis and/or mechanical thrombectomy. The study has a prospective randomized open blinded end-point (PROBE) (28) design.

### Selection/Treatment of Subjects

**Figure 1:**
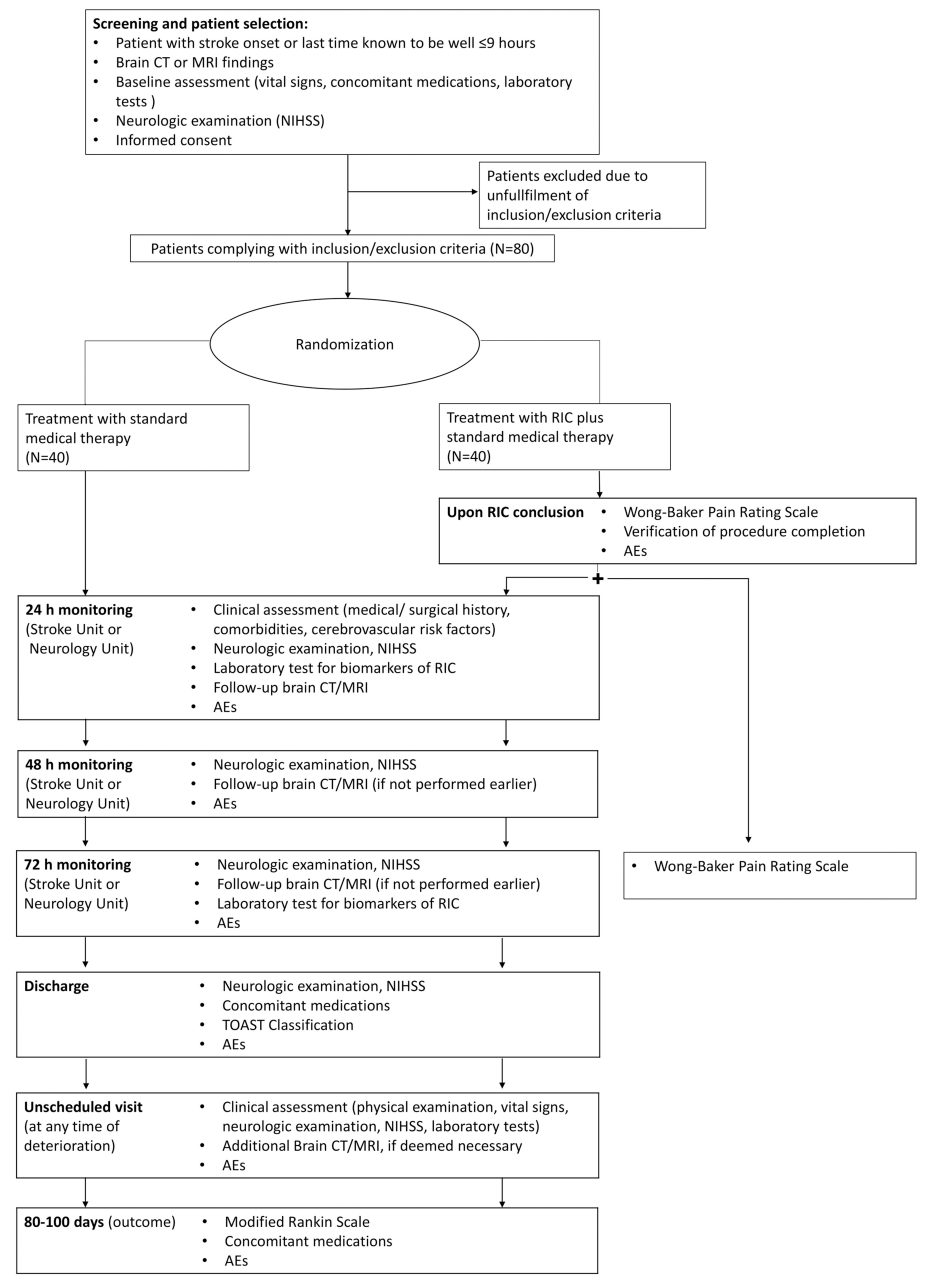
Flowchart of TRICS-9.

Patient enrollments (Figure 1) will be carried out in three ISO-associated academic hospitals provided with Comprehensive Stroke Centers of the Italian Hub-and-Spoke network for stroke. Patients will be eligible for inclusion in the trial if they have evidence of acute ischemic stroke due to either small or large vessel occlusions of the anterior circulation within 9 h of symptom onset, are 18 years of age or older, have a score on National Institutes of Health Stroke Scale (NIHSS; scores range from 0 to 42, with higher scores indicating a more severe deficit) equal to or higher than 5 and lower than 25, a pre-stroke score of 0 or 1 on the modified Rankin scale (which ranges from 0 to 6, with a score of 0 indicating no disability and higher scores indicating more severe disability), and patients or they surrogate provided informed consent. Strokes with unknown time of onset will also be considered for enrollment. For patients with stroke symptoms on awakening, the midpoint of sleep (i.e., the time between going to sleep and waking up with symptoms) will be considered as time of stroke onset and patients will undergo randomization if the estimated time of onset is ≤ 9 h (29); for patients with neurologic deficits (e.g., aphasia, anarthria, altered mental status) that keep them from reporting the time of onset, enrollment will be possible whenever the time elapsed since the patient was last known to be well is ≤ 9 h. Information regarding the time of going to sleep or the last time the patient was seen well will be obtained by the patient or anyone who last had contact with the patient before stroke onset. Patients eligible for either or both intravenous thrombolysis and mechanical thrombectomy will be excluded from the trial. Other exclusion criteria will be evidence of intracranial hemorrhage, vascular malformation, intracranial masses on brain CT or MRI, rapidly improving neurological symptoms at the time of first evaluation, transient ischemic attack with the resolution of symptoms at the time of first evaluation, amputation of the upper non-paretic arm, ulcers, phlebitis, or bad skin condition in the upper limbs, and history of peripheral artery disease or sickle cell disease. All inclusion and exclusion criteria are summarized in Table 1.

**Table 1:** Inclusion and exclusion criteria.

**Inclusion criteria**
• Clinical diagnosis and/or diagnosis on neuroimaging of anterior circulation acute ischemic stroke (due to either large or small vessel occlusion) within 9 h of symptom onset • Age ≥ 18 years • NIHSS ≥5 and <25 • Stroke with Unknown Time of Onset: this will include wake up strokes with estimated time of onset ≤ 9 h of enrollment or patients unable to report symptom onset but that were last known to be well ≤ 9 h of enrollment • Modified Rankin scale ≤ 2 prior to stroke onset • Written informed consent obtained from patient or legally responsible person
**Exclusion criteria**
• Candidates for intravenous thrombolysis and/or mechanical thrombectomy according to AHA/ASA guidelines • Brain CT or MRI detecting intracranial hemorrhage, vascular malformation, intracranial masses, or any medical condition unrelated to stroke that could explain symptoms • Rapidly improving neurological symptoms at the time of first evaluation • Transient ischemic attack • Amputation of the upper non-paretic arm • Ulcers or a bad skin condition in the upper limbs • History of peripheral artery disease, sickle cell disease, or upper limb phlebitis • Pregnancy • Ongoing participation in any interventional study • Terminal illness or life expectancy ≤ 6 months • Patients unable to give informed consent

*CT, computed tomography; MRI, magnetic resonance imaging; NIHSS, National Institutes of Health Stroke Scale*.

### Randomization

Participants will be randomly assigned to either control or experimental groups with a 1:1 allocation as per computer-generated block randomization schedule stratified by site. A specific “Randomization Procedure” document will be prepared, signed, and stored within a sealed envelope before the first randomization, The allocation sequence will be created by an individual separated from trial investigators and analysts. Treatment allocation will be disclosed after the patient has been recruited.

### Interventional Method

The experimental intervention will be performed in the Emergency Department immediately after randomization. A standard blood pressure cuff will be placed around the non-paretic arm. Patients randomized to RIC will undergo remote ischemic conditioning by four cycles of intermittent, manually induced, upper limb ischemia alternating 5 min of inflation (20 mmHg above the systolic blood pressure) to 5 min of deflation. Patients in the control group will have the blood pressure cuff placed around the non-paretic arm for 40 min but will undergo no inflations. All patients will receive usual care, at the discretion of the investigator and according to available guidelines and standards of care.

### Outcomes

Admission NIHSS will be assessed before randomization, whereas NIHSS at 72 h (NIHSS72) will be obtained by a neurologist blinded to treatment allocation.

The primary efficacy outcome will be early neurological improvement at 72 h, defined as percent change in NIHSS score [(Admission NIHSS—NIHSS72) × 100/Admission NIHSS]. This definition has been adopted in a recent study that re-assessed the primary outcome of the NINDS part 1 trial on intravenous thrombolysis, wherein percent change in NIHSS score resulted to be more sensitive in demonstrating early clinical benefit and more predictive of long-term neurological outcome (30, 31) than the absolute changes in NIHSS score.

Six secondary outcomes will be assessed: (1) NIHSS percent change at 24 h and 48 h: NIHSS will be assessed 24 h (NIHSS24) and 48 h after randomization (NIHSS48) and 2% changes will be independently calculated: [(Admission NIHSS—NIHSS24) × 100/Admission NIHSS] and [(Admission NIHSS—NIHSS48) × 100/Admission NIHSS]; (2) disability at 3 months, as assessed by the modified Rankin scale (mRS); (3) symptomatic intracerebral hemorrhage per the SITS-MOST definition: a local or remote Type 2 parenchymal hemorrhage on imaging 22–36 h after treatment or earlier if the imaging scan was performed due to clinical deterioration combined with a neurological deterioration of 4 NIHSS points from baseline or from the lowest NIHSS score between baseline and 24 h or leading to death within 24 h. A grading of Type 2 parenchymal hemorrhage for intracranial hemorrhage indicates a coagulum exceeding 30% of the infarct with substantial space occupation; (4) feasibility will be estimated by the proportion of patients randomized to a treatment group that complete the experimental intervention; (5) tolerability will be assessed by the Wong-Baker Faces Pain Rating Scale following the procedure and after 72 h; (6) an exploratory analysis on blood levels of HIF-1α and HSP27 24 h and 72 h after remote ischemic conditioning will be also carried out. Whole blood HIF-1alpha mRNA levels will be assessed at 24 and 72 h by quantitative reverse transcription polymerase chain reaction (HIF1a F, TCATCCAAG- GAGCCTTAACC; HIF-1a R, AAGCGACATAGTAGGGGCAC, Takara Bio, CA, USA). HIF-1alpha mRNA levels will be expressed as a ratio to the housekeeping gene GAPDH and the difference between HIF-1alpha mRNA levels at 24 h and 42 h will be defined as HIFDiff72 (HIF72 - HIF24). Plasma levels of HSP27 will be quantified using a colorimetric enzyme immunoassay (ELISA) kit (Enzo Life Sciences, Roma, Italy). The difference between plasma levels at 24 and 72 h will be expressed as HSPDiff72 (HSP72 - HSP24). An increase of HIF-1alpha and HSP27 levels at 72 h compared to 24 h is a biomarker of the peripheral vascular effect of RIC.

### Sample Size Estimates

In the NINDS trial, the NIHSS percent change in the control group was 15% +/– 35% at 24 h. The median of the NIHSS percent change in absence of treatment is expected to slightly increase, and the standard deviation to slightly decrease, at 72 h, due to stabilization of the neurological deficit. An estimated total sample size of 80 patients (40 patients in each arm) should yield 80% power to detect a clinically significant difference of 20% (40% in treatment vs. 20% in control arm) in the median percent change in NIHSS at 72 h, considering a standard deviation of 30%, at the two-sided statistical significance threshold of *p* = 0.05, when using a Wilcoxon-Mann-Whitney test. The primary null hypothesis is that there is no or negligible difference in clinical benefit between remote ischemic conditioning plus standard medical therapy and standard medical therapy alone.

### Data Monitoring Boards

An independent Data and Safety and Monitoring Board (DSMB) will be constituted by an experienced neurologist (E.Be.) of the Istituto di Ricerche Farmacologiche Mario Negri, a statistician (E.Bi.) of the Istituto di Ricerche Farmacologiche Mario Negri and a lay member (E.C.) of the Italian stroke patients' association A.L.I.C.E. Onlus (Associazione per la Lotta all'Ictus Cerebrale). The DSMB will provide expertise by periodical review and evaluation of the accumulated study data for participant safety, study conduct, and progress; it will monitor any serious adverse events, guarantee patient's dignity and fundamental rights with regard to treatments and care, and provide recommendations on continuation, modification, or termination of the trial.

### Data Analysis

The baseline patient characteristics of each treatment group will be described as means and standard deviations, medians, and quartiles, for numerical variables, and as frequencies and percentages per class for qualitative variables. Between-group unbalances among baseline characteristics will be considered for adjustment whenever deemed important for the outcome, in which case an adjusted secondary logistic analysis will be performed. The primary analysis of treatment effect on early neurological improvement will be performed using a Wilcoxon-Mann-Whitney test. Secondary analysis of the primary outcome will be performed using a mixed linear regression including treatment, centers, and any unbalanced baseline characteristics. Secondary outcomes will be analyzed using a Wilcoxon-Mann-Whitney test to evaluate treatment effect on early neurological improvement at 24 h or 48 h, and using a logistic mixed model to assess the effect of RIC on secondary variables (i.e., Wong-Baker face pain rating scale, modified Rankin scale, and occurrence of symptomatic intracerebral hemorrhage). The mRS scores will be dichotomized as a good functional outcome (mRS 0–2) and poor functional outcome (mRS 3–6). Wong-Baker Face Pain Rating Scale scores will be dichotomized around medians. Whole blood levels of HIF-1α mRNA and plasma levels of HSP27 in each arm will be represented by histograms. We expect no missing data for the primary outcome since patients will still be hospitalized. In case of missing results, a multiple imputation procedure will be used. Analyses will be conducted with an intention-to-treat approach (ITT). For the primary outcome, patients with neurological worsening will be given a percent change of 0; those deceased within 72 h will be given the last percent change available and a percent change of 0 will be assigned in case admission NIHSS is the only available data. A population of interest is composed of patients that completed the experimental intervention: a specific section will be devoted to this group, where main analyses will be repeated. The cut-off for statistical significance will be set at 0.05, two-tailed. Analyses will be carried out in blind using Stata/IC v. 15 or higher (Statacorp). A detailed Statistical Analysis Plan (SAP) will be written within 1 month after enrollment of the first patients.

### Allocation Concealment

This is an open label experimental study. Treatment allocation will be disclosed after patient recruitment, i.e., after inclusion/exclusion criteria have been recorded. Only outcome assessors (at 72 h and 3 months) and data analysts will be blinded after assignment to interventions.

### Study Organization and Funding

TRICS-9 is funded by a grant from the Italian Ministry of Health - PRIN 2017CY3J3W. This funding source had no role in study design and will have no role during its execution, analyses, interpretation of the data, reporting of the study, or decision to submit results.

## Discussion

The primary objective of the TRICS-9 trial is to assess the efficacy of RIC in patients with acute ischemic stroke that are not candidates for intravenous thrombolysis or mechanical thrombectomy due to ineligibility or contraindication, and that present to hospital within 9 h of symptom onset.

Previous clinical studies on the efficacy of RIC in acute ischemic stroke have provided neutral results (21, 22). A possible limitation of previous clinical trials was the inclusion of patients that received either or both intravenous thrombolysis and mechanical thrombectomy, since their efficacy may have concealed the effect of RIC. If RIC in combination with recanalization therapies appears to add no clinical benefit, whether it is beneficial to patients who are not candidates for recanalization therapies is still to be demonstrated. These patients represent a significant portion of those with acute ischemic stroke; indeed, despite progressive widening of eligibility criteria, the proportion of patients treated with recanalization therapies is limited (25, 26). TRICS-9 will involve patients that are not candidates for recanalization therapies due to ineligibility or contraindication and that may still benefit the neuroprotective action of RIC. Among these, common examples are (1) patients with distal occlusions with contraindications to intravenous thrombolysis because of ongoing anticoagulant treatment, elevate blood pressure despite the administration of blood pressure lowering medications, recent surgery or trauma or previous intracranial hemorrhage; (2) patients with lacunar strokes with contraindications to intravenous thrombolysis due to stroke onset beyond 4.5 h and insufficient mismatch ratio on perfusion imaging; (3) patients with large vessel occlusion with early infarct signs on brain imaging and insufficient mismatch ratio to be a candidate for thrombectomy, but who may still exhibit some salvageable brain tissue on perfusion studies. We decided to exclude mild strokes (NIHSS <5) due to the propensity they show toward spontaneous favorable neurologic outcomes, and those with very severe strokes (NIHSS ≥25). The choice to select such a population has two advantages: (1) it offers RIC to patients that otherwise would not be offered any acute treatment and would receive only secondary prevention, and (2) it tests the efficacy of RIC in acute stroke patients without the confounding factor represented by recanalization therapies that might mask the benefit of RIC.

The 9-h time window has been inspired by recent clinical trials on recanalization therapies in acute ischemic stroke, such as EXTEND and WAKE-UP trials, that demonstrated potentially salvageable tissue up to 7–10 h from symptom onset, even in patients without large vessel occlusion (29, 32). The neuroprotective effect of RIC is presumably advantageous in presence of salvageable brain tissue, whose survival can last many hours after stroke onset depending on multiple factors. Many preclinical studies indicate that RIC helps the survival of salvageable brain tissue by reducing apoptosis, downregulating inflammatory response, and improving collateral circulation in the ischemic penumbra (33). Furthermore, two pre-clinical studies on a rat model of experimental ischemic stroke demonstrated that RIC is beneficial up to 6 h after ischemic stroke due to permanent large vessel occlusion (18, 19). Based on this evidence, we hypothesize that the application of RIC may be beneficial up to several hours after stroke onset in presence of salvageable brain tissue.

In a previous clinical trial (23), wherein penumbral salvage was the primary outcome and both infarct growth and final infarct size were secondary outcomes, no between-group differences were found in patients treated with RIC plus standard therapy and in those that received standard therapy alone. However, radiological findings represent surrogate outcomes allowing only indirect measurement of treatment efficacy and the lack of demonstration of a positive radiologic effect does not exclude a clinical benefit with certainty. Taking a different approach, in TRICS-9 a clinical outcome has been chosen to obtain a direct measure of RIC efficacy.

The primary outcome will be early neurological improvement, defined as NIHSS percent change at 72 h, following the definition adopted in a recent study that re-assessed the NINDS part 1 trial on intravenous thrombolysis in acute ischemic stroke (30, 31). This primary outcome has many advantages: (I) it accounts for the wide variation in admission scores across patients, (II) it is sensitive enough to allow a higher study power than that achievable using absolute changes in the NIHSS score, even with a relatively small sample size, (III) it is sufficiently hard to test a large effect size of immediate clinical interest, and (IV) the study power associated with a continuous outcome variable is expected to be higher than that achieved using a dichotomous outcome variable.

In a previous study (23), RIC was applied by emergency medical services to patients with suspected stroke during transfer to hospital. Although application shortly after symptom onset is reasonable in view of a prompt activation of neuroprotective mechanisms, many stroke mimics were initially recruited. In our trial, patient selection will be carried out in the emergency department only after neurologic examination and radiologic investigations, to prevent the inclusion of stroke mimics.

RIC has been investigated in many studies; however, the underlying mechanisms remain elusive and no reliable biomarkers are available. Despite the observation of HIF-1α and HSP27 to be associated with RIC, there is no solid evidence of their role as mediators. HSP27 is neuroprotective against cerebral infarction when purified from human lymphocytes and injected in mice models (5, 6), as well as when overexpressed in mice models of ischemic stroke (7). A significant increase in serum total HSP27 and phosphorylated HSP27 (pHSP27) was detected in stroke patients 4 days after RIC compared to controls (16). HIF-1α is involved in neuroprotection against ischemic brain injury (8–10), and RIC promotes HIF-1a activation in the peripheral blood (11, 12). These observations suggest HSP27 and HIF1α to be good candidates as biomarkers. Hence, an exploratory analysis on blood levels of HSP27 and HIF1-α will be performed to assess their potential role as biomarkers of neuroprotection following RIC.

This trial has limitations. First, the expected effect size of RIC in TRICS-9 was inspired by evidence from experimental studies on *in vivo* models of ischemic stroke (13, 15) that demonstrated an absolute difference of about 50% in the infarct size of rats with transient middle cerebral artery occlusion treated with RIC as compared to those untreated. However, the expected effect magnitude of RIC in TRIC-9 has been voluntarily set at 20% to reduce the risk of an overambitious outcome, resulting in smaller effect size than those observed in other experimental studies. If TRICS-9 will provide neutral results, it will not exclude that RIC may determine a milder clinical effect than that considered in this trial, in which case further dedicated studies will be necessary: in any case, it will provide valuable information for a potential phase III study. Second, the sample size is relatively small; however, the study design is adequately powered to detect an early neurological improvement, and both the characteristics of the highly selected study population and recruitment rate have been taken into account. Third, RIC will be carried out manually rather than automatically, which may implicate heterogeneity in manoeuver execution; however, the manoeuver is not supposed to be significantly influenced by the operator, since the interventional procedure is intentionally simple and will be performed by dedicated and instructed personnel. Fourth, the choice of a highly selected patient population could reduce the probability of recruitment; however, to maximize the probability of recruitment, only Comprehensive Stroke Centers, wherein a high influx of patients is expected, will be involved in the study.

## Ethics Statement

The studies involving human participants were reviewed and approved by Comitato Etico ASST Monza. The patients/participants provided their written informed consent to participate in this study.

## Author Contributions

SB conceived the study and initiated the study design. SD contributed to study design and helped with its implementation. MT provided expertise in clinical trial design. CF is the grant holder. All authors contributed to the refinement of the study protocol and approved the final manuscript.

## Conflict of Interest

The authors declare that the research was conducted in the absence of any commercial or financial relationships that could be construed as a potential conflict of interest. The handling editor is currently organizing a Research Topic with one of the authors SB.

## Publisher's Note

All claims expressed in this article are solely those of the authors and do not necessarily represent those of their affiliated organizations, or those of the publisher, the editors and the reviewers. Any product that may be evaluated in this article, or claim that may be made by its manufacturer, is not guaranteed or endorsed by the publisher.
